# Evaluation of the Effectiveness of the National Clean Air Programme in Terms of Health Impacts from Exposure to PM2.5 and NO_2_ Concentrations in Poland

**DOI:** 10.3390/ijerph20010530

**Published:** 2022-12-28

**Authors:** Paulina Jagiełło, Joanna Struzewska, Grzegorz Jeleniewicz, Jacek W. Kamiński

**Affiliations:** 1 Institute of Environmental Protection—National Research Institute, 00-548 Warsaw, Poland; 2 Faculty of Building Services, Hydro and Environmental Engineering, Warsaw University of Technology, 00-661 Warsaw, Poland

**Keywords:** air pollution, PM2.5, NO_2_, air quality modelling, health effects in Poland, AirQ+

## Abstract

The health impact of air quality has recently become an emerging issue. Many regions, especially densely populated regions, have deteriorating air quality. The primary source of pollution in Poland is the municipal sector. Air pollutants have a negative impact on human health, contribute to premature deaths, and are the cause of various diseases. Over recent years, Europe’s air quality has largely improved due to several measures to reduce pollutant emissions. The following paper presents the impact of annual average PM2.5 and NO_2_ concentrations on premature deaths in Poland in 2019. Exposure to PM2.5 caused more than 19 000 premature deaths, and exposure to NO_2_ contributed to around 6000 premature deaths in 2019. Taking 2019 as a baseline, the impact of the envisaged implementation of the national Clean Air Programme on the number of premature deaths is analysed. Implementing the mitigation measures focused on replacing inefficient stoves and boilers in individual households would significantly improve air quality, mainly particulate matter. Reducing PM2.5 concentrations would reduce premature deaths by around 3000 cases, while for NO_2,_ the number of premature deaths would not change much.

## 1. Introduction

The impact of air pollution on human health has become an important issue in recent years. The number of studies on the impact of air pollution concentrations on increased mortality, cardiovascular diseases, or respiratory diseases is increasing yearly [1]. This provides a more accurate estimate of health impacts from exposure to air pollution in different regions.

Air pollution is one of the leading causes of mortality and morbidity worldwide [2,3]. Children and the elderly are particularly vulnerable to air-pollution-related health outcomes [4,5,6]. A significant proportion of air pollution is generated by the combustion of fossil fuels [7]. In Poland, over 50% of PM2.5 and PM10 emissions originate from coal and wood combustion processes in households that are released near the ground level [8]. Combustion processes in power generation and industry contribute to the long-distance transport of pollutants and background concentrations. Residential sector emissions result in elevated local concentrations of primary pollutants, especially during wintertime stagnant weather conditions [9,10,11]. Consequently, increased concentrations of pollutants cause negative health effects, mainly related to the respiratory tract.

Many research studies support the link between exposure to air pollution and adverse health outcomes [12]. Long-term and short-term exposure to air pollution contributes to an increased risk of premature deaths, the incidence of disease, or Potential Years of Life Lost (PYLL). Air pollution is estimated to have contributed to 4 million deaths worldwide in 2017 [13].

According to the EEA’s 2021 report [14], in 27 European countries, the estimated number of premature deaths due to PM2.5 concentrations was approximately 307,000 cases, and 40,400 cases due to NO_2_ concentrations in 2019. The total number of PYLL in 2019 attributed to PM2.5 exposure in the 27 European countries was 4,068.000 years, and 512,800 years due to NO_2_. Additionally, it was reported that a downward trend from previous years was observed. However, Poland remains one of Europe’s countries with the highest health impacts from exposure to PM2.5 concentrations. The report states that in 2019 in Poland, the number of premature deaths due to PM2.5 concentrations was estimated at more than 39,300 cases, and the annual average NO_2_ concentration contributed to 1190 cases. In addition, the years of life lost were estimated at more than 490,000 years across the country. 

Additionally, another analysis of the premature mortality rate due to air pollution in 969 European cities found high exposure in Poland [15]. In 2015, 8% of all deaths could have been avoided in the Silesia Agglomeration if the annual average PM2.5 concentrations had been lower than the WHO recommendation (10 µg/m^3^ for PM2.5). As many as 3 Polish cities in the Silesia voivodeships ranked in the top 10 with the highest health impacts from exposure to poor air quality [16,17].

Most of the analysis refers to the WHO guidelines for air quality standards published in 2005 [18]. The air quality standards were updated in 2021 [19]. The threshold concentrations of pollutants have been tightened. The annual average concentration of PM2.5 has been reduced from 10 µg/m^3^ to 5 µg/m^3^, PM10 from 20 µg/m^3^ to 15 µg/m^3^, and NO_2_ from 40 µg/m^3^ to 10 µg/m^3^. Consequently, the extent of areas not meeting air quality thresholds postulated by WHO has increased significantly.

Over recent years, air pollutant emissions in the European Union have shown a clear downward trend [20]. Between 2000 and 2017, total emissions in the 28 EU countries fell by 31% for PM2.5, 29% for PM10, 46% for NO_x_, and 80% for SO_x_. Despite the apparent decreases in emissions, air pollutant concentrations in European cities exceed recommended standards. For example, in 2019, the annual average concentration of PM2.5: Leskovac in Serbia (26.16 µg/m^3^), Plovdiv in Bulgaria (27.19 µg/m^3^), Nazilli in Turkey (27.68 µg/m^3^), and Krakow (26.52 µg/m^3^) and Katowice (26.84 µg/m^3^) in Poland [21]. The marked reduction in pollutant emissions has also contributed to an evident decline in health impacts from exposures to air pollution, with the number of premature deaths due to PM2.5 decreasing by more than 4.5% (per 1 million inhabitants) between 2000 and 2017. This is due to effectively reducing pollutant emissions and implementing measures to improve air quality. 

Poland has a significant problem with air quality compared to other European countries. Poland’s main air quality issue is PM10 and PM2.5, as well as benzo(a)pyrene. Permissible EU standards are exceeded mainly in the southern part of Poland (Silesia and Małopolska voivodeships) and in larger cities. The main cause is emission from the municipal and household sector and combustion of solid fuels, very often of poor quality. In 2019 air quality standards were exceeded at many monitoring sites, especially in the south of Poland [22].

To improve air quality, a national Clean Air Programme was initiated in 2019 [23]. The main goal of the programme is to reduce or avoid emissions of particulate matter and other pollutants into the atmosphere, mainly from single-family dwellings, by improving the energy efficiency of buildings, with particular emphasis on replacing old stoves and boilers with modern, lower-emission types. The programme is based on receiving financial assistance to partially cover the costs of replacing the heat source. The programme will operate for 10 years until the end of 2029. The anticipated final effect is a reduction in residential emissions of PM10 and PM2.5, and other pollutants compared to 2017.

This paper aimed to estimate the health impacts of exposure to PM2.5 and NO_2_ concentrations in 2019 in Poland and to show how the partial implementation of the national Clean Air Programme could reduce health impacts from exposure depending on the two scenarios of emission reduction measures. The World Health Organisation model AirQ+ (The Air Quality Health Impact Assessment Tool) was used to estimate the health effects of air pollution exposure [24].

## 2. Materials and Methods

### 2.1. Study Area

**Figure 1:**
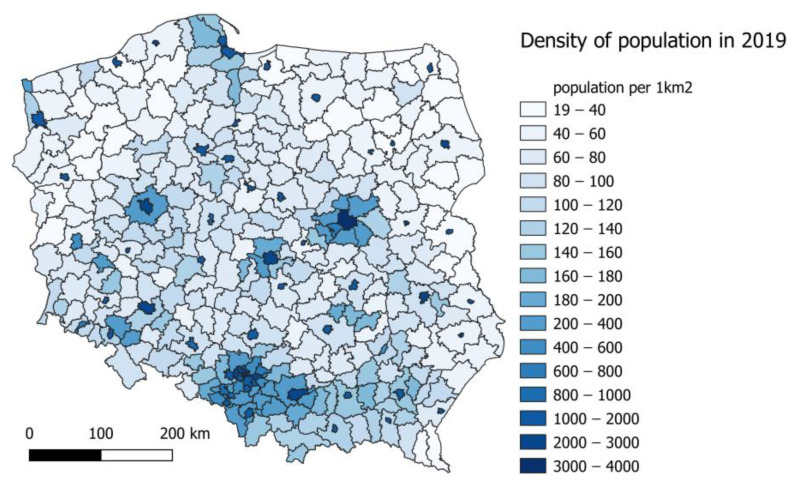
Population density per country in Poland in 2019.

The following analysis covers the area of Poland (Figure 1). The area of Poland covers 322,575 km^2^ and lies between 49° and 54°50′ north latitude and between 14°07′ and 24°09′ east longitude. In 2019, the population was 38,383,000, while the population density was 123 persons per 1 km^2^. However, the variation in population density between counties is large. Modelling results used in this study were calculated with a spatial resolution of ~2.5 km. Health impact assessment analyses were performed for 380 counties in 2019.

### 2.2. GEM-AQ Model

The pollutants concentration fields used in this study were calculated in support of the air quality policy in Poland (Environmental Protection Act, Art 66, paragraph 6). The national air quality modelling system based on the GEM-AQ model [25] was used for this study.

GEM-AQ is a semi-Lagrangian chemical weather model in which air quality processes (chemistry and aerosols) and tropospheric chemistry are implemented online in a weather prediction model, the Global Environmental Multiscale (GEM) [26] model, which was developed at Environment Canada. The gas-phase chemistry mechanism used in the GEM-AQ model is based on a modified version of the Acid Deposition and Oxidants Model (ADOM) [27], where additional reactions in the free troposphere were included. Aerosol processes are represented by parameters of nucleation, coagulation, and intra-cloud processes, including liquid phase chemistry for sulphur compounds and leaching inside the cloud, as well as sedimentation and dry and wet deposition. Transport processes include advection, turbulence diffusion, and deep convection. Mass distribution is represented in 12 aerosol particle size ranges describing the logarithmic increase in particle radius. The modelled values of PM10 and PM2.5 concentrations are calculated as the sum of respective fractions of individual chemical components. The model has been evaluated in several research projects [28,29,30,31].

Since 2018 the model has been used for the national air quality forecast and assessment for the Chief Inspectorate of Environmental Protection and operates as a partner model in the Copernicus Atmosphere Monitoring Service—Regional Production (CAMS2_40) [32].

Emission data for Poland are stored in the Central Emission Database, compiled using a high-resolution bottom-up inventory developed and maintained by the National Centre for Emission Management. For the energy production sector and industry, the data are based on annual reporting by facilities. The methodology for residential emission modelling is described in [33]. Annual emissions are transformed into monthly emission rates using weighting factors from annual emission profiles. Emission profiles are assigned to Standard Nomenclature for Air Pollution (SNAP) categories [34]. 

Computations were performed using a 0.025-degree resolution grid. The GEM-AQ model is set up to perform calculations using 28 vertical layers. The lower 21 layers are in the troposphere. Hourly output of PM2.5 and NO_2_ concentrations from the surface layer were used to calculate annual averages for further analysis.

### 2.3. Scenarios

Health impacts from exposure to air pollution were estimated based on three scenarios: baseline and two versions of measures applied to emissions from the residential sector. The baseline scenario is based on the pollutant concentrations in 2019 [35], while scenarios 1 and 2 examine the change in pollutant concentrations assuming a reduction in emissions from municipal and household sources due to the partial implementation of the national Clean Air Programme. New gas or fuel oil boilers will have to meet requirements of energy efficiency class at least A as defined in the Commission’s Delegated Regulation (EU) NR 811/2013 of 18 February 2013 and Regulation (EU) 2017/1369 of the European Parliament and of the Council of 4 July 2017, setting a framework for energy labelling and repealing the Directive 2010/30/EU [36]. The legal basis for the programme is the regulation to support thermomodernisation and renovation and the central register of emissivity of buildings of 21 November 2008 (with further amendments) [37].

The national Clean Air Programme envisages that 3 million existing single-family buildings and 1 million new buildings will undergo thermal modernisation or replacement of obsolete solid fuel heat sources with modern boilers meeting the highest standards. 

**Figure 2:**
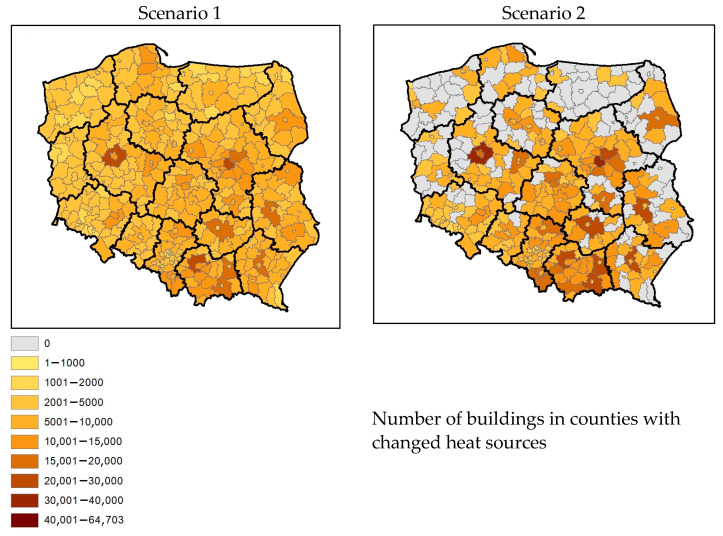
Number of buildings in counties with changed heat sources in scenarios 1 and 2.

Since the programme uptake is lower than expected, we have addressed partial implementation (50%) and focused only on the replacement of old solid fuel heat sources. This allowed for precise calculations of the primary emission changes. At the same time, in the case of building insulations, estimating heat demand and assumptions in the heating installation would introduce high uncertainty of the emission scenario. In the proposed scenarios, it was assumed that half of the buildings, or approximately 2 million detached houses, would have the heat source replaced. In detached houses (BDOT code 1110—Database of Topographical Objects—single-family buildings) [38], the emission factors were changed to correspond to the fuel standards of modern heating installations. The number of buildings in the counties with changed heat sources is shown in Figure 2. Buildings were randomly selected in areas of a county meeting certain conditions based on modelling results for the 2019 assessment:

- Base scenario—emissions from the national Central Emission Database for 2019 without modifications.
- Scenario 1—emission reductions were applied in all voivodeships in Poland. Since there are approx. 6.628 million single-family buildings in Poland, the change of emission factors was implemented for 30% of buildings in each administrative unit.
- Scenario 2—emission reductions were applied in 254 administrative units (out of 380), where the modelled annual average PM2.5 exceeded 20 µg/m^3^ in 2019. The change was applied to 39% of buildings in each selected county. For this scenario, if an exceedance occurred, even in one model grid, the entire county was eligible for emission reductions.

**Figure 3:**
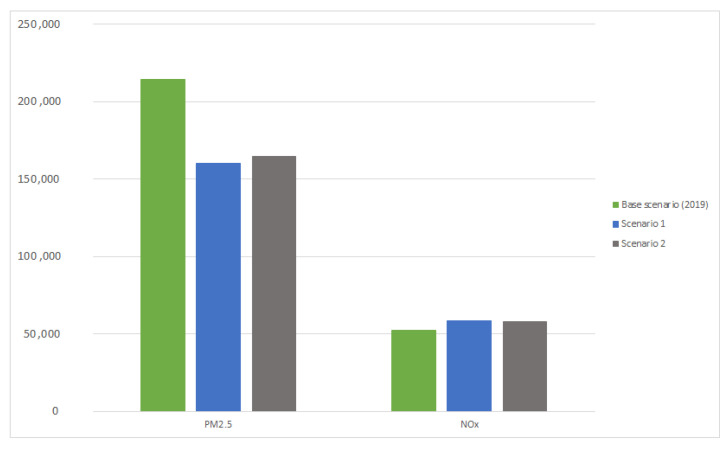
Total emissions of PM2.5 and NO_2_ in base scenario, scenario 1, and scenario 2.

In the baseline scenario, total PM2.5 emissions were assumed to be 214,067 Mg/year, and NOx emissions 52,018 Mg/year (Figure 3). The sum of PM2.5 emissions in scenario 1 was reduced to 160,245 Mg/year and in scenario 2 to 164,806 Mg/year. For NOx, total emissions in scenario 1 were 58,357 Mg/year, and in scenario 2, 57,778 Mg/year. In the first and second scenarios, emission reductions affected 2 million detached buildings. However, in scenario 1, this applied to buildings across Poland, and in scenario 2, only to buildings in counties with exceedances of the permissible average PM2.5 concentration (20 µg/m^3^).

For both scenarios, the coal and wood combustion emission factors adopted for the Central Emission Database were changed to those adopted in the National Air Pollution Control Programme (NAPCP) [39] for the estimation of the reduction of air pollutants in the municipal and household sector for buildings covered by the Clean Air Programme. According to the assumptions, the projections proposed wider use of gas fuel-base stoves, which increased the emissions of NOx. 2.4 AirQ+ Model.

The AirQ+ Model (The Air Quality Health Impact Assessment Tool), developed by the WHO, is used to assess health risks associated with air pollution and is a widely used tool to estimate the possible health impact of air quality [40]. AirQ+ estimates the effects of air pollutants on human health in two aspects: mortality (the number of premature deaths caused by exposure to high pollutant concentrations) and morbidity (how much of the morbidity (for example, pneumonia) is caused by high pollutant concentrations). Health effects can be estimated for the following pollutants: PM2.5 and PM10, NO_2_, ozone, and black carbon.

The evaluation of the AirQ+ is based on the Relative Risk (RR) estimation that reflects the magnitude of an association between exposure and disease. RR indicates the likelihood of developing the disease in the exposed group, PDE, relative to those who are not exposed, PDU. This likelihood is equal to PDE/PDU. Relative Risks due to air pollution are modelled with the function,

(1) $$RR=exp^{\beta \left(x-x_{0}\right)}$$

where *x* is the pollutant concentration (μg/m^3^), and *x_0_* is the cut-off threshold; for example, the background concentration or the lowest possible value is (μg/m^3^). In the log-linear model, *β* denotes the change in the *RR* for a one-unit change in concentration *x* [41].

The AirQ+ model is a frequently used tool for estimating the health impacts of pollutants. In Poland, for example, it has been used to assess the effects of PM2.5 and PM10 dust in Wrocław [42] and 12 other agglomerations between 2008 and 2018 [43]. The software is also widely used for estimations in other countries, for example, Pakistan [44], Bosnia and Herzegovina [45], Iraq [46], Iran [47], and Spain [48].

### 2.4. Methodology and Data

In the AirQ+ model, the basic information required to estimate health impacts from exposure is the population’s annual average pollutant concentrations and mortality statistics. In the presented analyses, the following information was used:

- Annual average concentrations of PM2.5 and NO_2_, calculated using the GEM-AQ model for the reference scenario 2019, averaged over counties;
- Annual average concentrations of PM2.5 and NO_2_ obtained in scenarios 1 and 2, using the GEM-AQ model, averaged over counties;
- Number of deaths per county in 2019, according to the Central Statistical Office [49];
- The total population in counties in 2019, according to the Statistical Office [49];
- Relative risk factor, assumed based on the HRAPIE project [50], as recommended by the WHO.

The assumed relative risk and the reference concentration (cut-off threshold) above which health impacts from exposure were estimated are shown in Table 1. Population and deaths for scenarios 1 and 2 were assumed as in 2019. Reference concentrations were assumed at 10 µg/m^3^. Although the WHO guidelines for air quality standards state that any concentration of PM2.5 and NO_2_ impacts human health, the level of 10 µg/m^3^ can be treated as a representative of the natural background plus the transboundary transport over Poland.

## 3. Results

The GEM-AQ modelling results were averaged over the modelling period to obtain gridded annual concentration fields and further averaged over administrative regions.

### 3.1. Air Quality Changes

**Figure 4:**
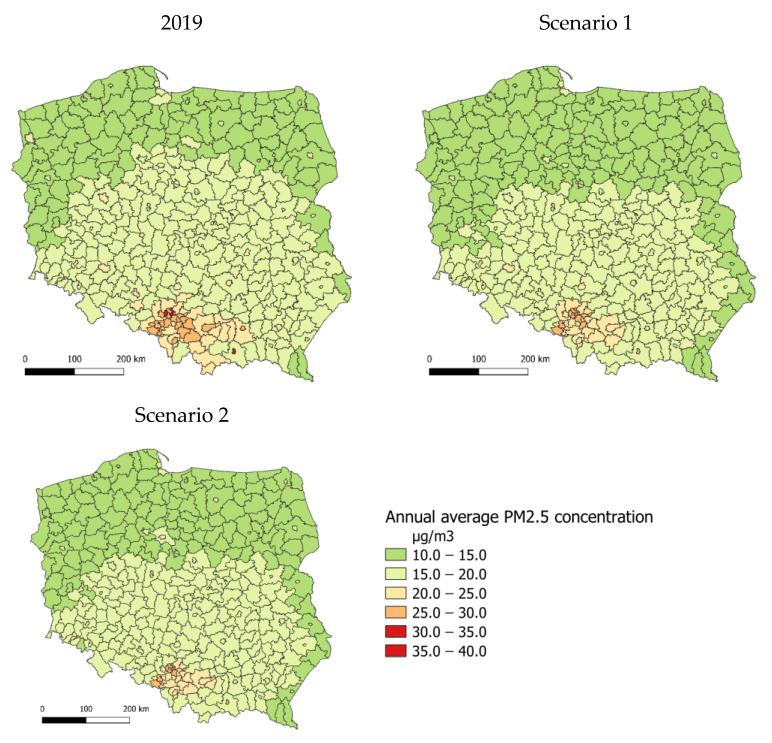
Spatial distribution of annual average PM2.5 concentrations in 2019 and for scenarios 1 and 2 (county average).

Figure 4 shows the annual average concentrations of PM2.5 in 2019 for scenarios 1 and 2. In 2019, the annual average concentrations of PM2.5 in counties ranged from 12 to 34 µg/m^3^. Exceedances of the permissible concentration level (20 µg/m^3^) occurred in major cities throughout the country, particularly in southern Poland. The results for scenario 1 show that more counties in the central and eastern parts of the country would be characterised by the annual average PM2.5 concentrations in the lower range—from 10 to 15 µg/m^3^. Concentrations throughout the country would range from 12 to 29.5 µg/m^3^. In scenario 2, the distribution of annual average concentrations has a similar pattern.

**Figure 5:**
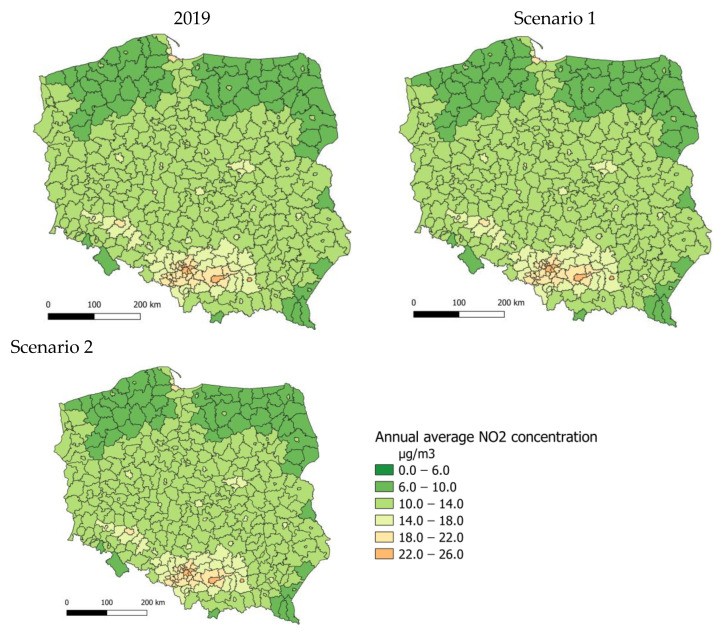
Spatial distribution of annual average NO_2_ concentrations in 2019 and for scenarios 1 and 2 (county average).

Figure 5 shows the spatial distribution of annual average NO_2_ concentrations in 2019 for scenarios 1 and 2. In 2019, annual average nitrogen dioxide concentrations calculated for the counties ranged from 8 to 24 µg/m^3^. Higher concentrations occurred in the central and southern parts of Poland. The assumed change in emissions in scenarios 1 and 2 would result in a slight increase in NO_2_ concentrations across the country. The spatial distribution is similar for the base and two analysed scenarios.

### 3.2. Health Effects

**Figure 6:**
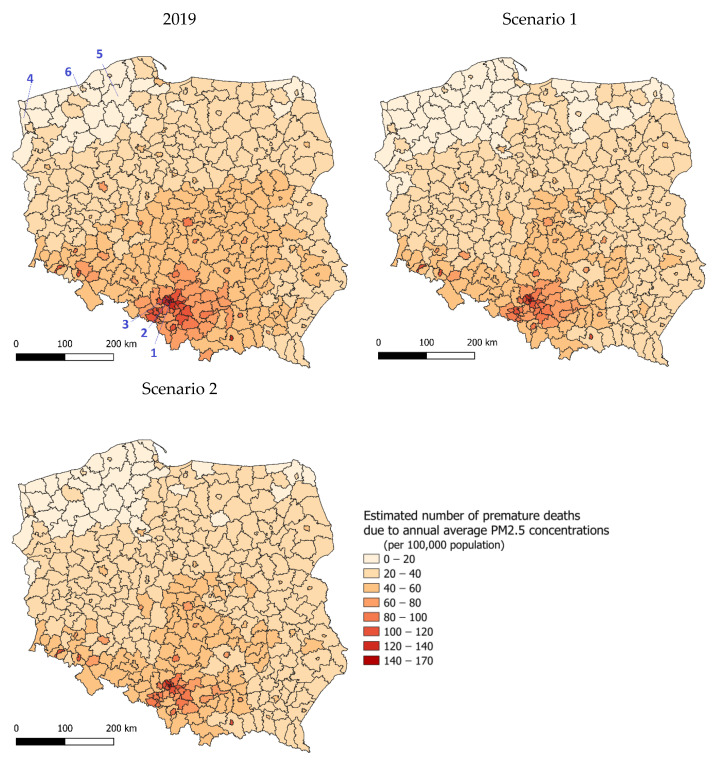
Estimated number of premature deaths due to annual average PM2.5 concentrations in 2019, as projected for scenario 1 and scenario 2; per 100,000 population; counties averages. Numbers 1–6 refer to the counties presented in detail in Table 2.

In 2019, premature deaths in all counties varied from 11 to 167 cases per 100,000 inhabitants. Lower values occurred in the northwestern and the northeastern parts of the country. Notably, higher values occurred in the area of the Silesia and Małopolska voivodeships (Figure 6). The emission changes assumed in scenarios 1 and 2 and a resulting decrease in PM2.5 concentrations led to a large reduction in exposure-related health impacts. The number of counties with the lowest number of premature deaths, between 0 and 20, would increase in the north of the country. Table 2 compares three counties with the highest and lowest exposure for the base and two emission scenarios.

**Figure 7:**
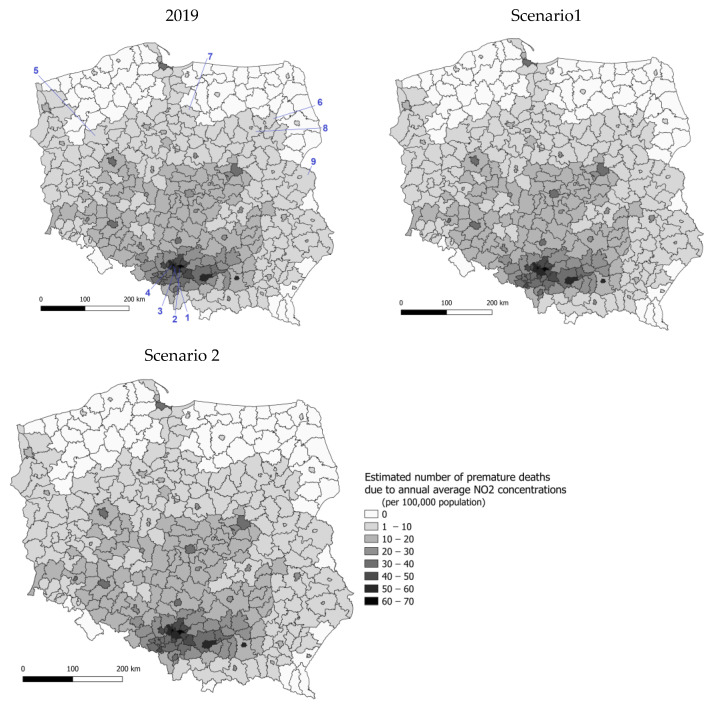
Estimated number of premature deaths due to annual average NO_2_ concentrations in 2019, as projected for Scenario 1 and Scenario 2; per 100 000 population; counties averages. Numbers 1–9 refer to the counties presented in Table 3.

Figure 7 shows the estimated number of premature deaths due to the annual average NO_2_ concentrations in 2019, scenarios 1 and 2. In 2019, the estimated number of premature deaths due to NO_2_ ranged from 0 to 70 cases per 100,000 inhabitants. Lower exposure occurred in the northern and eastern parts of Poland. In comparison, higher exposure occurred in the central and southern voivodeships (Silesia and Małopolska). Emission changes in scenarios 1 and 2 would result in a slight increase in NO_2_-concentration-related mortality. The spatial distribution would be very similar to the pattern calculated for 2019. Table 3 shows three counties with the highest exposure, three with the lowest exposure for the base, and two emission scenarios. In all counties where the annual average NO_2_ concentration was below 10 µg/m^3^, the health impacts from exposure are equal to 0 due to the assumed reference level.

## 4. Discussion

**Figure 8:**
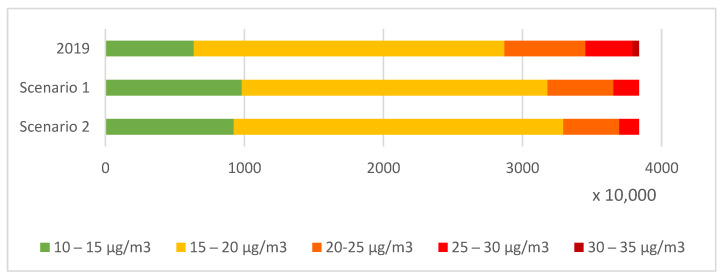
Population exposed to annual average concentrations of PM2.5 in Poland in 2019 and for scenario 1 and scenario 2.

**Figure 9:**
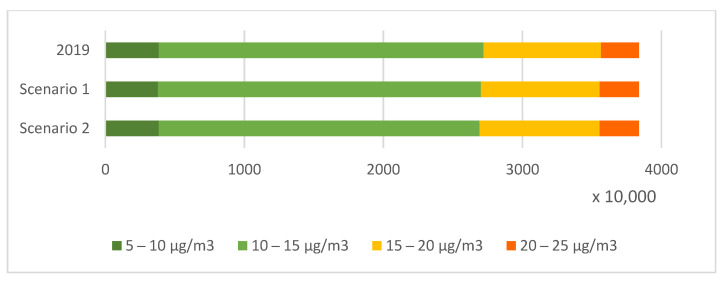
Population exposed to annual average concentrations of NO_2_ in Poland in 2019 and for scenario 1 and scenario 2.

Figure 8 and Figure 9 show the total number of people exposed to annual average concentrations of PM2.5 and NO_2_ in 2019 and the changes due to emission reduction assumed in scenarios 1 and 2. For PM2.5, annual concentration below 10 µg/m^3^ averaged for the counties did not occur in 2019. Most of the population lived in areas where the annual average PM2.5 concentration ranged from 15 to 20 µg/m^3^. However, about 25% of the population in 2019 lived in places where annual average concentrations exceeded the 20 µg/m^3^ threshold. The change in emissions assumed in scenario 1 would reduce the population exposed to the annual average concentrations exceeding the permissible level to 17%. In scenario 2, with the measures applied in areas with the highest exposure, the population exposed would be about 14%.

In contrast, the lowest annual NO_2_ concentration averaged over the counties ranged from 5 to 10 µg/m^3^. Most of the population lived in areas where concentrations were from 10 to 15 µg/m^3^. The limit level of 40 µg/m^3^ was not exceeded. The assumed changes in scenarios 1 and 2 would not relevantly change the number of people exposed.

Our analysis estimated that 19,332 premature deaths occurred nationwide in 2019 due to annual average PM2.5 concentrations, accounting for 5% of all deaths in 2019 (Table 4). Implementation of the national Clean Air Programme under scenario 1 would reduce the number of premature deaths to 16,464 cases, accounting for 4.3% of all deaths. The emissions change in scenario 2 would result in 16,324 premature deaths due to annual average PM2.5 concentrations, representing 4.3% of all deaths. The assumed emission changes in scenario 2, i.e., focusing air quality improvement measures on most counties with the highest PM2.5 emissions, would reduce health exposures slightly more than in scenario 1. Relative to 2019, the reduction in premature deaths would be around 15% for both scenarios.

In 2019, the estimated number of premature deaths due to annual average nitrogen dioxide concentrations was 6008 nationwide. This represented 1.5% of all deaths in 2019. Assumed emission changes in scenario 1 and scenario 2 would result in a slight increase in the number of premature deaths to 6092 cases in scenario 1 and 6094 cases in scenario 2 (Table 5).

The difference in PM2.5 emissions between the scenarios assumed for the partial implementation of the national Clean Air Programme and the baseline was approximately 50,000 Mg/year. The average annual concentration of PM2.5 in Poland would be reduced by about 1.1 µg/m^3^. Depending on the county, the reduction ranged from 0.23 µg/m^3^ to 4.43 µg/m^3^. The decrease in concentrations reduced the estimated total number of premature deaths due to annual average PM2.5 concentrations by about 3000 cases. A small increase in NOx emissions would not result in relevant changes in analysed health effects.

According to reports from the European Environment Agency (EEA), estimates of premature deaths due to PM2.5 concentrations in Poland are doubled compared to the estimates presented in this analysis and the analysis undertaken for 2018 [51]. This is because of the different reference concentrations above which health impacts from exposure are calculated. In our work, this level is set at 10 µg/m^3^. In comparison, EEA estimates are based on PM2.5 concentrations above 0 µg/m^3^. Health impacts from exposure estimates are also much higher in other works based on the EEA methodology [52,53]. The different results of the health effect also depend on the relative risk factor adopted [54].

**Figure 10:**
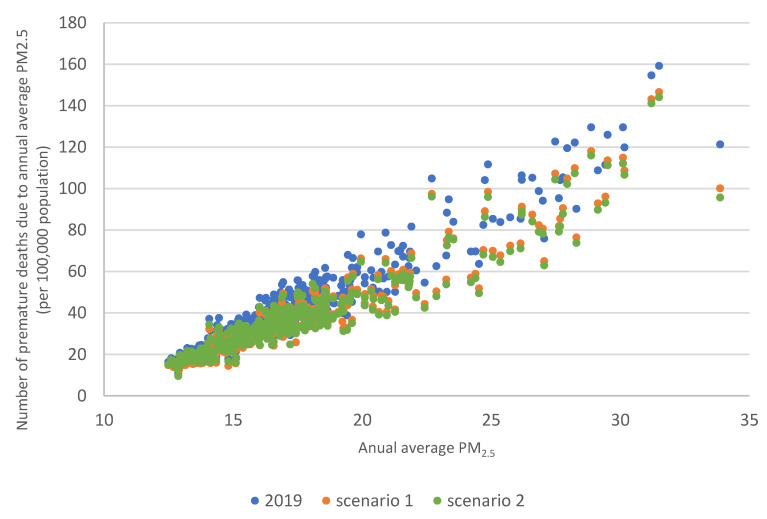
Number of premature deaths per 100 000 inhabitants due to high PM2.5 concentrations in counties in Poland in 2019 (blue points) and scenario 1 (orange points) and scenario 2 (green points).

**Figure 11:**
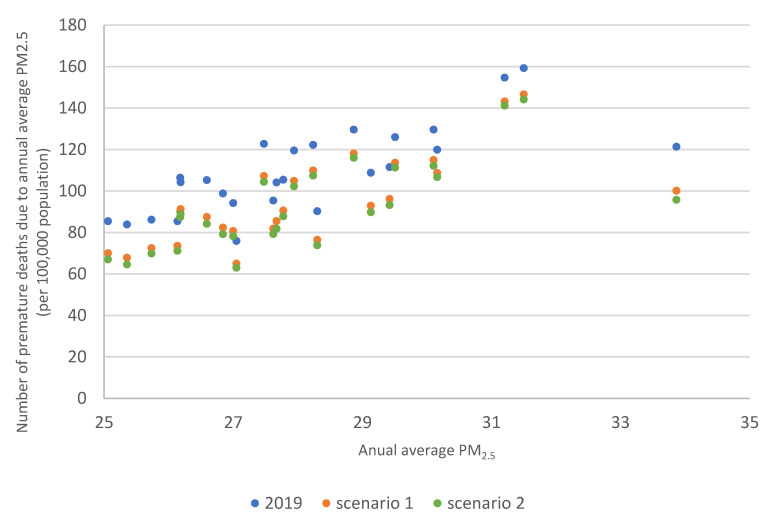
Number of premature deaths per 100,000 inhabitants due to PM2.5 concentrations above 25 µg/m^3^ in counties in Poland in 2019 (blue points) and scenario 1 (orange points) and scenario 2 (green points).

Scatterplots 10 and 11 show the relationship between the annual average PM2.5 concentration and the number of premature deaths in the baseline scenario 2019 and scenarios 1 and 2. Figure 10 is for all counties in Poland, and Figure 11 is for counties where the annual average PM2.5 concentration was higher than 25 µg/m^3^. Figure 10 shows that the relationship is close to linear. The correlation factor for 2019 was: 0.96 for scenario 1:0.95 and scenario 2:0.94. In Figure 11, the average reduction in premature deaths between 2019 and scenario 1 was 14 cases (highest reduction—21cases, lowest reduction—11 cases), and between 2019 and scenario 2, an average of 17 cases (highest reduction—26 cases, lowest reduction—13 cases). Emission changes in scenario 2 would result in higher reductions in premature deaths in counties where annual average PM2.5 concentrations are higher than 25 µg/m^3^.

## 5. Conclusions

The health effects were analysed based on annual average concentrations of PM2.5 and NO_2_. The total number of premature deaths was estimated due to the annual average concentration of PM2.5 and NO_2_. The aim was to calculate the changes in health impacts from exposure due to the measures assumed as a partial implementation of the national Clean Air Programme in Poland. The number of premature deaths was calculated for 2019 and two emission scenarios.

In 2019, the estimated number of premature deaths due to annual average PM2.5 concentrations was 19,332 cases countrywide. The assumed emission reductions in both scenarios would reduce the number of premature deaths to 16,464 cases in scenario 1 and 16,324 cases in scenario 2. This would represent approximately 15% fewer premature deaths relative to 2019.

In 2019, the estimated number of premature deaths due to annual average nitrogen dioxide concentrations was 6008 cases nationwide. Projections based on assumed emission changes in scenario 1 and scenario 2 showed a slight increase in premature deaths to approximately 6 100 cases for both scenarios (scenario 1—6092, scenario 2—6094).

The highest exposure for all scenarios occurred in counties in Silesia and Małopolska voivodeships. These are mainly: the city of Chorzów, Świętochłowice, Bytom Piekary Śląskie, Ruda Śląska, Sosnowiec, and Nowy Sącz.

The study aimed to assess the health impacts of exposures in 2019 and which course of action to improve air quality would result in higher reductions in premature deaths. Even partial implementation of the national Clean Air Programme would improve air quality, and negative health effects of PM2.5 concentrations would be lower. A comparison of the change in health impacts from exposure estimated from emission reductions in scenario 1, which assumed country measures, with scenario 2, which took remediation targeted at areas with the highest concentrations, shows that slightly higher reductions would occur under scenario 2. Focusing emission reduction activities on areas with the worst air quality would reduce health impacts from exposure. However, the scenario dedicated to vulnerable areas did not bring significant improvements compared to the scenario with proportional implementation throughout the country.

## Figures and Tables

**Table 1:** Assumed relative risks and reference concentrations for the health impacts from exposure analyses performed.

Pollution	Health Exposure	Relative Risk [RR]	Reference Concentration [µg/m^3^]
PM2.5	Number of premature deaths from natural causes	1.062	10
NO_2_	Number of premature deaths from natural causes	1.041	10

**Table 2:** Counties with the highest estimated number of premature deaths due to annual average PM2.5 concentrations (per 100,000 population) in 2019 and based on scenario 1 and scenario 2 forecasts. The counties are shown on the map (Figure 5) numbered from 1 to 6.

			2019		Scenario 1		Scenario 2	
			Counties	Premature Deaths (Per 100,000 Inhabitants) Populations	Counties	Premature Deaths (Per 100,000 Inhabitants)	Counties	Premature Deaths (Per 100,000 Inhabitants)
Estimated number of premature deaths due to annual average PM2.5 concentrations (per 100,000 population)	Highest exposure	1	Chorzów	160 107,807	Chorzów	147	Chorzów	144
		2	Świętochłowice	155 49,557	Świętochłowice	143	Świętochłowice	141
		3	Bytom	130 165,263	Bytom	118	Bytom	116
	Lowest exposure	4	Policki	10 80,652	Policki	10	Policki	10
		5	Bytowski	15 79,198	Bytowski	13	Koszaliński	14
		6	Koszaliński	16 66,480	Koszaliński	14	Bytowski	14

**Table 3:** Counties with the highest estimated number of premature deaths due to annual average NO_2_ concentrations (per 100,000 population) in 2019 and based on scenarios 1 and 2 forecast. The counties are shown on the map (Figure 7) numbered 1 to 9.

			2019		Scenario 1			Scenario 2		
			Counties	Premature Deaths (Per 100, 000 Inhabitants) Populations	Counties		Premature Deaths (Per 100,000 Inhabitants) Populations	Counties		Premature Deaths (Per 100,000 Inhabitants) Populations
Estimated number of premature deaths due to annual average NO_2_ concentrations (per 100,000 population)	Highest exposure	1	Chorzów	66 107,807		Chorzów	67		Chorzów	67
		2	Sosnowiec	58 199,974		Sosnowiec	59		Sosnowiec	59
		3	Katowice	56 292,774		Katowice	57	4	Świętochłowice	57 49,557
	Lowest exposure	5	Czarnkowsko-Trzcianecki	0.4 86,990		Czarnkowsko-Trzcianecki	0.4		Czarnkowsko-Trzcianecki	0.4
		6	Łomżyński	0.4 50,943	8	Ostrołęcki	0.4 88,654		Ostrołęcki	0.4
		7	Nowomiejski	0.4 43,822	9	Bialski	0.5 110,772		Bialski	0.5

**Table 4:** Estimated number of premature deaths nationally due to annual average PM2.5 concentrations in 2019 and based forecasts on scenario 1 and scenario 2.

	2019	Scenario 1	Scenario 2
Estimated number of premature deaths due to annual average PM2.5 concentrations (Reference concentration 10 µg/m^3^) The number of natural deaths	19,332 383,909	16 464	16 324
Percentage of premature deaths nationally due to annual average PM2.5 concentrations	5%	4.3%	4.3%

**Table 5:** Estimated number of premature deaths nationally due to annual average NO_2_ concentrations in 2019 and based forecasts on scenario 1 and scenario 2.

	2019	Scenario 1	Scenario 2
Estimated number of premature deaths due to annual average NO_2_ concentrations (Reference concentration 10 µg/m^3^) The number of natural deaths	6008 383,909	6092	6 094
Percentage of premature deaths nationally due to annual average NO_2_ concentrations	1.5%	1.55%	1.55%

## Data Availability

Research data are not shared.
